# Lyme borreliosis in Finland: a register-based linkage study

**DOI:** 10.1186/s12879-020-05555-w

**Published:** 2020-11-10

**Authors:** Eeva Feuth, Mikko Virtanen, Otto Helve, Jukka Hytönen, Jussi Sane

**Affiliations:** 1 Institute of Biomedicine, University of Turku, Kiinamyllynkatu 10, FI-20521 Turku, Finland; 2 Department of Internal Medicine, Turku University Hospital, Kiinamyllynkatu 4–8, FI-20521 Turku, Finland; 3 Department of Health Security, Finnish Institute for Health and Welfare, PO Box 30, FI-00271 Helsinki, Finland; 4 Clinical Microbiology, Turku University Hospital, Kiinamyllynkatu 10, FI-20521 Turku, Finland

**Keywords:** Lyme borreliosis, Epidemiology, Surveillance, Finland, Register-based study

## Abstract

**Background:**

In Finland, the routine surveillance of Lyme borreliosis (LB) is laboratory-based. In addition, we have well established national health care registers where countrywide data from patient visits in public health care units are collected. In our previous study based on these registers, we reported an increasing incidence of both microbiologically confirmed and clinically diagnosed LB cases in Finland during the past years. Here, we evaluated our register data, refined LB incidence estimates provided in our previous study, and evaluated treatment practices considering LB in the primary health care.

**Methods:**

Three national health care registers were used. The Register for Primary Health Care Visits (Avohilmo) and the National Hospital Discharge Register (Hilmo) collect physician-recorded data from the outpatient and inpatient health care visits, respectively, whereas the National Infectious Diseases Register (NIDR) represents positive findings in LB diagnostics notified electronically by microbiological laboratories. We used a personal identification number in register-linkage to identify LB cases on an individual level in the study year 2014. In addition, antibiotic purchase data was retrieved from the Finnish Social Insurance Institution in order to evaluate the LB treatment practices in the primary health care in Finland.

**Results:**

Avohilmo was found to be useful in monitoring clinically diagnosed LB (i.e. erythema migrans (EM) infections), whereas Hilmo did not add much value next to existing laboratory-based surveillance of disseminated LB. However, Hilmo gave valuable information about uncertainties related to physician-based surveillance of disseminated LB and the total annual number of EM infections in our country. Antibiotic purchases associated with the LB-related outpatient visits in the primary health care indicated a good compliance with the recommended treatment guidelines.

**Conclusions:**

Avohilmo and laboratory-based NIDR together are useful in monitoring LB incidence in Finland. A good compliance was observed with the recommended treatment guidelines of clinically diagnosed LB in the primary health care. In 2018, Avohilmo was introduced in the routine surveillance of LB in Finland next to laboratory-based surveillance of disseminated LB.

## Background

Lyme borreliosis (LB) is caused by the spirochetes of *Borrelia burgdorferi* sensu lato complex and is transmitted to humans via a tick bite [1]. The first and most common manifestation of LB is erythema migrans (EM). The diagnosis of typical EM is clinical and when detected, antibiotic treatment without any laboratory testing should be initiated [2]. Based on available evidence of randomized clinical trials, oral antibiotics for 2–3 weeks are recommended for treating EM [3]. In Finland, EM is typically diagnosed and treated by general practitioners (GP) in the primary health care, whereas disseminated LB, such as Lyme neuroborreliosis (LNB), is treated in the hospitals or outpatient clinics of hospitals under the counselling of infectious diseases specialists.

In Europe, the highest LB incidences are reported in central and northern Europe, and in Baltic countries [4, 5]. Several studies have shown an increasing incidence in LB cases during the past two decades [5–10]. However, the comparison of actual, national incidence rates among countries is difficult due to lack of uniform and standardized diagnostic methods, case definitions, reporting practices, and surveillance systems. Only some European countries have a mandatory notification system for LB [7, 8, 11–13]. In those countries, LB surveillance is mostly based on laboratory surveillance resulting in that the clinically diagnosed EM is greatly neglected. In the countries without mandatory notification, LB incidence estimates are based on epidemiological studies conducted often in high-risk population or in high-endemic areas, thus not representing the burden of LB in general population nor in the whole country [14–16].

In Finland, routine surveillance of LB has been laboratory-based since 1995. In addition, we have well established national health care registers where countrywide data from patient visits in public health care units are collected. In our previous study, we investigated the epidemiology of LB in Finland for the period 1995–2014 by using the data of these health care registers [17]. During this period, the incidence of microbiologically confirmed disseminated LB cases increased around 4–5-fold from 7/100,000 population to 31/100,000 population. During 2011–2014, the incidence of clinically diagnosed LB cases increased as well and reached the highest incidence of 61/100,000 population in 2014.

With the aim to further evaluate and validate our register data and to refine LB incidence estimates provided in our previous study, we used a personal identification number in register-linkage to identify LB cases on an individual level. In addition, we evaluated treatment practices considering LB in the primary health care by investigating antibiotic purchases associated with LB cases in the Register for Primary Health Care Visits (Avohilmo).

## Methods

### The national health care system

In Finland (population 5.5 million), 16 geographically and administratively defined hospital districts (HD) provide primary and secondary health care services, and five are also responsible for tertiary care services (Technical appendix Figure 1 in [17]). The autonomous region of the Åland Islands provides primary and secondary health care and is considered as the 21st HD. All patients in need of non-acute medical care are primarily assigned to the GPs working in the primary health care units, whereas a proportion of people seeks medical help from the private health care. In acute illness, the patient can be admitted to the hospital without a referral from a GP or private health care physician.

### The health care registers

In order to assess the total LB case numbers in Finland (clinically diagnosed EM infections and microbiologically confirmed disseminated LB infections), data of three national health care registers, the Register for Primary Health Care Visits (Avohilmo), the National Hospital Discharge Register (Hilmo), and the National Infectious Disease Register (NIDR), were reviewed. These health care registers are maintained by the National Institute for Health and Welfare (NIHW).

LB cases in Avohilmo and Hilmo represent patient visits in outpatient (municipal health centers) and inpatient (hospitals) health care units, respectively. These health care visits are notified to the registers by the consulting physician. Notifications include the basic information of the patient (personal identification number, age, sex), the detailed information of the health care service obtained (the place of health care unit, the reason for physician consultation, investigations, and treatment), and the discharge diagnoses according to the International Classification of Diseases, revision 10 (ICD-10). LB cases are notified by the ICD-10 code “A69.2”. Positive findings in LB diagnostics (serological or molecular confirmation) are electronically notified to NIDR by the microbiological laboratories on a routine basis. Thus, LB cases in NIDR mostly represent disseminated LB infections and are here referred to as “microbiologically confirmed LB cases”. The contents of Avohilmo, Hilmo, and NIDR, and LB case definitions are described in more detail in [17].

For this study, the year 2014 was chosen as a case study year. LB cases from Avohilmo and Hilmo were retrieved by the ICD-10 code “A69.2”, and all microbiologically confirmed LB cases were extracted from NIDR. Any entry in the register during 2014 was included regardless of the mutual chronological order of the events in the different registers. However, only one entry per personal identification number was included. The personal identification number was used to link the LB cases in the different registers on an individual level. Descriptive statistics was performed including analyses on counts and frequencies using Microsoft Excel 2013 (Redmond, WA, USA).

### The patient records

The patient records of those LB cases reported to Hilmo in 2014, but which were not microbiologically confirmed (i.e. the cases that could not be linked to NIDR by personal identification number), were more thoroughly reviewed. These cases are here referred to as “microbiologically unconfirmed hospital cases”. The aim was both to characterize the LB cases that were diagnosed in inpatient health care units but that were not microbiologically confirmed, and to evaluate the quality of Hilmo concerning LB notifications. We focused on two main hospital districts (HD) in Finland, Helsinki and Uusimaa HD (population 1.6 million) and Varsinais-Suomi HD (population 480,000), which locate in the highly endemic LB area in south coast of Finland [17]. Thus, a subgroup of microbiologically unconfirmed hospital cases (i.e. all cases in these two main hospital districts during 2014) was evaluated.

### Antibiotic purchase data

In order to evaluate the treatment practices considering LB in the primary health care, we used the personal identification number to link the antibiotic purchases with the LB cases notified to Avohilmo during 2014. The antibiotic purchase data was obtained from the Finnish Social Insurance Institution that maintains a national database on purchases and reimbursement payments of prescribed medicines [18]. Our focus was on the antibiotic treatment practices of the clinically diagnosed LB (i.e. EM) in the primary health care, and therefore, the analysis did not include the evaluation of treatment practices of microbiologically confirmed, disseminated LB in the hospital setting.

## Results

In the study year 2014, altogether 3628 LB cases were identified in Avohilmo, 1420 in Hilmo, and 1696 in NIDR.

### Evaluation of clinically diagnosed (Avohilmo) LB cases

**Fig. 1:**
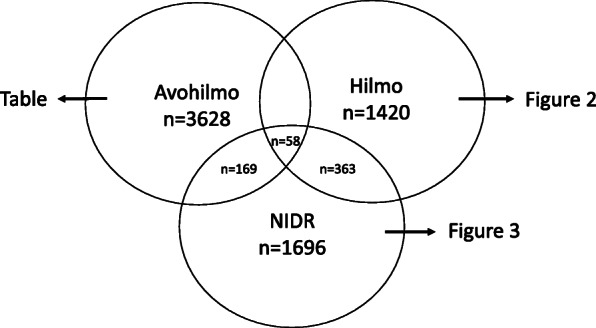
Total number of Lyme borreliosis cases in each national health care register, Finland, 2014. 227 Avohilmo cases and 421 Hilmo cases were microbiologically confirmed i.e. the cases were also found in NIDR. 58 LB cases were in all three registers. Avohilmo = The Register for Primary Health Care Visits, Hilmo = National Hospital Discharge Register, NIDR = National Infectious Diseases Register

Out of 3628 LB cases identified in Avohilmo, 227 (6.3%) were also found in NIDR, i.e. they were microbiologically confirmed (Fig. 1). However, the vast majority (93.7%) of the LB cases identified in Avohilmo were not microbiologically confirmed and thus, most likely represent the clinically diagnosed LB cases i.e. EM infections. The share of those LB cases in Avohilmo that were microbiologically confirmed stayed fundamentally the same over the age-groups (0–9, 10–19, 20–29 years, and so forth) and HDs indicating that the age or the place of primary care consultation did not significantly affect the likelihood of EM/LB to be confirmed microbiologically (data not shown).

### Evaluation of hospital discharge-based (Hilmo) LB cases

**Fig. 2:**
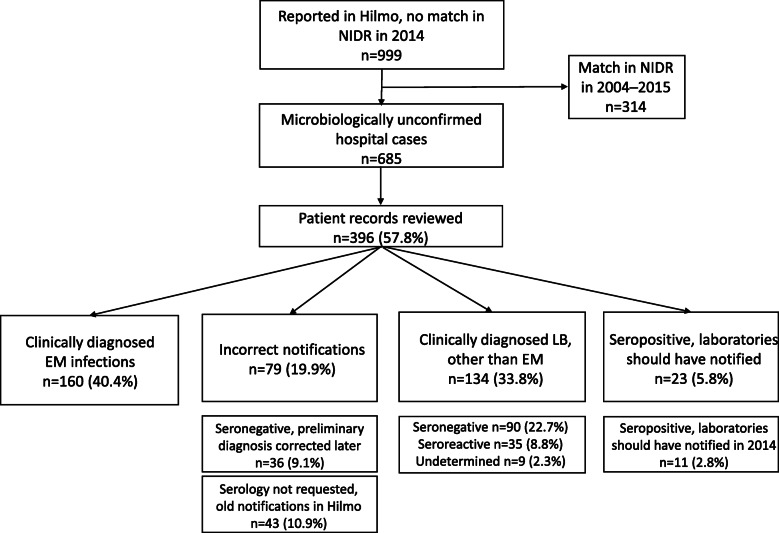
Lyme borreliosis cases in Hilmo that were not microbiologically confirmed in 2014. 314 additional cases could be extracted from NIDR when the linkage-time was relaxed to the 2004–2015 period. These include Hilmo entries where microbiological testing for LB was performed years earlier than 2014 but where the diagnoses were repeated in subsequent hospital visits without a suspicion of a new infection (“old LB notifications in Hilmo”), and Hilmo entries where microbiological testing was performed either just at the end of 2013 or at the beginning of 2015 i.e. hospital cases that truly represent new LB infections in 2014. Patient records of two major hospital districts, Varsinais-Suomi HD and Helsinki and Uusimaa HD, representing 57.8% of microbiologically unconfirmed hospital cases in 2014, were reviewed. EM = Erythema migrans, Hilmo = National Hospital Discharge Register, LB = Lyme Borreliosis, NIDR = National Infectious Diseases Register

Out of 1420 LB cases in Hilmo, 421 (29.6%) were microbiologically confirmed and thus, also found in NIDR (Fig. 1). 999 LB cases (70.4%) in Hilmo could not be linked to NIDR (Fig. 2). When the linkage time was relaxed to the 2004–2015 period, 314 more cases could be extracted from NIDR resulting in remaining 685 (48.2%) cases notified in Hilmo as “LB” without the microbiological confirmation.

To further characterize these microbiologically unconfirmed hospital cases, patient records of the subgroup of 396 (57.8%) cases were reviewed one at a time. Out of those, 160 (40.4%) represented EM infections that were clinically diagnosed in the hospitals and thus, no microbiological testing was performed (Fig. 2). 79 (19.9%) cases were judged incorrect notifications in Hilmo after careful reviewing. Almost half of these entries (36 out of 79) originated for example from the outpatient hospital clinics where LB suspicion was notified to Hilmo as a preliminary diagnosis but after borrelia serology turned out to be negative, the case was no longer considered as LB, some other ICD-10 code than “A69.2” was notified and the patient did not receive antimicrobial treatment for LB. Of the other half (43 out of 79) of these “incorrect notifications” laboratory testing for LB was not requested at all in the study year 2014. Most of these cases were old LB notifications in Hilmo which kept on re-notifying in the patient records along with subsequent hospital visits. 134 (33.8%) cases were seronegative, borderline reactive, or the serological result could not be determined in the study year 2014 but these cases were still notified to Hilmo as LB possibly based solely on the strong clinical suspicion of LB (Fig. 2: “Clinically diagnosed LB, other than EM”). In contrast to the seronegative cases among “incorrect notifications”, most of these patients received antibiotic treatment for LB. 23 (5.8%) cases were clearly seropositive at the time of investigation and thus, should have been electronically notified to NIDR by the laboratories. However, in fact only 11 (2.8%) were positive in 2014 and thus, were missed in our study year. Age-group or HD did not significantly affect the probability of LB case in Hilmo to be microbiologically confirmed (data not shown).

### Evaluation of microbiologically confirmed (NIDR) LB cases

**Fig. 3:**
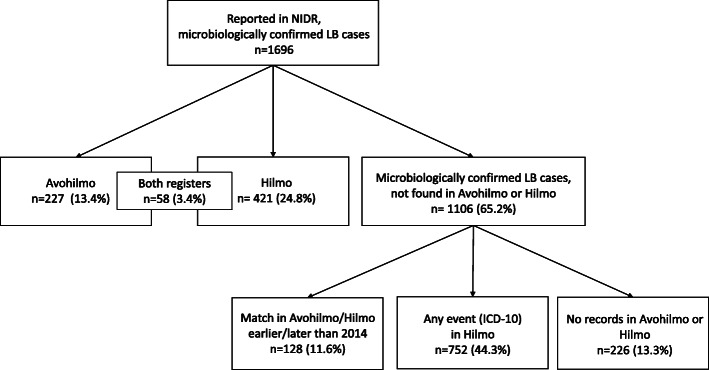
The distribution of microbiologically confirmed Lyme borreliosis cases among Avohilmo and Hilmo. “Any event (ICD-10) in Hilmo” refers to microbiologically confirmed LB cases that were notified to Hilmo with any other ICD-10 code except “A69.2”. Most of the microbiologically confirmed LB cases in NIDR that could not be linked to any records in Avohilmo or Hilmo were probably diagnosed in the occupational or private health care. Avohilmo = The Register for Primary Health Care Visits, Hilmo = National Hospital Discharge Register, ICD-10 = International Classification of Diseases, revision 10, LB = Lyme borreliosis, NIDR = National Infectious Diseases Register

Each microbiologically confirmed LB case in NIDR should have a physician remittance and thus, an entry of the health care consultation should be found in Avohilmo, Hilmo, occupational or private health care. Out of 1696 microbiologically confirmed LB cases in NIDR, 227 cases (13.4%) were found in Avohilmo and 421 (24.8%) in Hilmo (Fig. 1). 58 LB cases (3.4%) were in both registers. Thus, 1106 (65.2%) microbiologically confirmed LB cases in NIDR could not be linked to primary health care or to hospital event in 2014 (Fig. 3). However, out of these, 128 (11.6%) cases were notified in Avohilmo or Hilmo at the end of 2013 or in the beginning of 2015. Despite the positive serological or molecular finding in LB diagnostics, 752 (44.3%) LB cases were notified in Hilmo with other ICD-10 code than “A69.2”. When the ICD-10 entries related to these cases were further evaluated, no systematic error in the use of ICD-10 codes suggesting that one ICD-10 code would have been used more often than another, was identified. Out of the rest 226 (13.3%) cases, no entry of the health care consultation was found in Avohilmo or Hilmo.

### Evaluation of treatment practices considering clinically diagnosed LB cases in the primary health care

Altogether, 3386 (93.3%) of the outpatient visits due to LB in the primary health care in 2014 lead to antibiotic purchase (Table 1). Amoxicillin was the most often purchased antibiotic in 3046 (90.0%) cases. Doxycycline was purchased in 1332 (39.3%) cases, and azithromycin in 366 (10.8%) cases. The distribution of antibiotic purchases was rather similar over the HDs, and there were no clear differences among the age groups except that doxycycline was uncommonly used antibiotic in children under 9 years of age (data not shown). In this age group, amoxicillin was by far the most commonly used antibiotic.

**Table 1:** Purchased antibiotics after primary health care visits due to Lyme borreliosis, Finland, 2014

	Number of cases	Percent
**Total number of LB cases in Avohilmo**	3628	100
Microbiologically confirmed LB cases	227	6.3
Clinically diagnosed LB cases	3401	93.7
LB cases that lead to antibiotic purchase	3386	93.3
**Antibiotic purchase**		
Amoxicillin	3046	90.0
Doxycycline	1332	39.3
Azithromycin	366	10.8

## Discussion

Some European countries, including Finland, notify microbiologically confirmed LB cases routinely but clinically diagnosed EM is greatly neglected. In our previous study, we evaluated the incidence and the geographic distribution of LB in Finland by using three national health care registers: Avohilmo, Hilmo, and NIDR [17]. Here, we used a personal identification number in order to link individual LB cases in these registers, and thereby to evaluate our register data and to refine our previous LB incidence estimates. Avohilmo was found to be useful in monitoring clinically diagnosed EM cases, whereas Hilmo did not add much value next to current laboratory-based surveillance of disseminated LB. However, Hilmo revealed some shortcomings related to physician-based surveillance and improved the estimation of the total annual number of EM infections in Finland.

In our previous study, we evaluated the total annual number of LB cases in Finland (6440 cases in 2014) by summing clinically diagnosed and microbiologically confirmed LB cases together [17]. This calculation included an assumption that the number of clinically diagnosed LB cases in Avohilmo does not substantially overlap with the number of microbiologically confirmed cases in NIDR. Here, the overlap between Avohilmo and NIDR registers was 6.3% in 2014 indicating that, indeed, the vast majority of the LB cases identified in Avohilmo represent clinically diagnosed EM infections. Since no laboratory testing for EM is either required or recommended, EM cases are not found in NIDR. Those 227 cases notified to both registers, NIDR and Avohilmo, presumably represent disseminated LB cases diagnosed in the primary health care and/or early seroconverted EM infections where laboratory tests were performed despite the recommendations. However, this rate of overlapping between Avohilmo and NIDR is so small that the double-counting of LB cases should not cause major bias to the estimated total number of LB cases in Finland. The number of clinically diagnosed EM cases where laboratory tests were performed and returned seronegative is unknown and would be of future research interest in order to understand the laboratory test ordering practices of physicians in the primary health care. In the recently published study from Norway, the antibody testing was performed in ~ 20.0% of GP diagnosed EM cases [19].

Next to laboratory-based reporting of LB, the inclusion of EM as another key indicator has been suggested to LB surveillance in Europe [20, 21]. Some European countries have estimated the incidence of LB and the frequency of its different manifestations through repeated cross-sectional physician surveys or through register-based studies monitoring of notified in- and outpatient diagnoses [9, 13, 14, 16, 22]. In these studies, the provided EM incidence estimates rarely represent the whole country or all the physicians involved in the diagnostics and treatment of LB within the country. In contrast, Avohilmo is countrywide and involves all GPs in the public primary health care. When a compulsory ICD-10 code is entered in the patient record after GP consultation, LB case is notified to Avohilmo automatically without the need for active reporting by the physician. Some underestimation of the number of clinically diagnosed LB cases is, however, acknowledged since Avohilmo does not yet cover occupational or private health care visits. Moreover, subjective clinical misjudgment by the physician may lead to under- or over-reporting of clinically diagnosed LB cases although typical EM is most likely well recognized in Finland. Despite these shortcomings, Avohilmo appeared to be a useful tool in monitoring EM infections in Finland.

According to the international and national guidelines, the first line treatment for EM is oral amoxicillin [3, 23]. Doxycycline can be used for patients who are allergic to penicillin, but if contraindicated (e.g. in children < 8 years and in pregnancy), azithromycin is the alternative. In this study, the high overall percentage of antibiotic purchases (93.3%), together with the fact that amoxicillin and doxycycline were the most often purchased antibiotics for clinically diagnosed LB, indicate a good compliance with the recommended treatment guidelines. In a recently published Norwegian study, the most often prescribed antibiotic for EM in general practice was phenoxymethylpenicillin [19]. However, phenoxymethylpenicillin is not recommended for EM in Finnish guidelines. The total number of antibiotic purchases proportioned to the LB cases in Avohilmo exceeded 100% most likely because some of the patients have been prescribed other antimicrobial subgroups –perhaps due to side-effects– before finishing the first treatment. A proportion of patients may have received a second course of antibiotics after the first one although prolonged or repeated treatment is not recommended for LB [24]. Here, we investigated which antibiotic regimens were chosen for the treatment of clinically diagnosed LB. In order to investigate whether the prescribed dosing and the duration of the treatment for clinically diagnosed LB also follow the recommended guidelines, more detailed data from the Finnish Social Insurance Institution is needed in the future.

Because Hilmo contains nationwide linkable data on hospital discharges, we expected that LB cases in Hilmo represent mainly disseminated LB infections. Such cases should be microbiologically confirmed and hence, almost every LB case in Hilmo should have an entry found in NIDR. However, surprisingly, over two thirds of the LB cases identified in Hilmo (999 out of 1420 cases) were not microbiologically confirmed (Fig. 2). When a wider time period 2004–2015 was used in NIDR, 685 cases remained without microbiological confirmation. Most of the microbiologically unconfirmed hospital cases were notified to NIDR years earlier which means that all the Hilmo entries in 2014 were not new infections during the same year. The same was noted after evaluating the patient records of the subgroup of microbiologically unconfirmed hospital cases (*n* = 396) in 2014. Around 10% of those Hilmo cases were old notifications in the register, where microbiological testing for LB was performed years earlier, but the diagnoses were repeated in subsequent hospital visits without a suspicion of a new infection. These cases were never notified to NIDR by the laboratories because the microbiological result at the time of investigation had been either negative or borderline reactive. In addition to old notifications, some over-reporting of the total annual number of LB cases in Hilmo is caused by the so-called working diagnoses (suspected LB) notified before the microbiological confirmation was obtained. However, < 10% of analysed microbiologically unconfirmed hospital cases were eventually reported by other ICD-10 code than “A69.2”.

In 2014, laboratories missed to notify 2.8% of positive LB findings in the subgroup of microbiologically unconfirmed hospital cases. More under-reporting of LB cases in inpatient hospital setting occurs due to insufficient reporting practices by physicians which is reflected by the fact that 44.3% of 1696 microbiologically confirmed LB cases in 2014 were reported in Hilmo with other ICD10-codes than “A69.2” (Fig. 3). These cases probably represent true LB cases where the physicians missed entering ICD-10 code for LB to the patient records. Similarly, a recently published Swedish study found that less than half of all microbiologically confirmed LNB patients received the recommended combination of ICD-10 codes for LNB and only two thirds of the patients received the code for LB at all [13].

All in all, Hilmo seems to be a heterogenic and imperfect register and it does not add much value next to NIDR to estimate the number of disseminated LB infections in Finland. Both over- and under-reporting of LB cases occur due to above-mentioned reasons related to reporting practices. On the other hand, it gives valuable information considering the total number of EM infections in our country. Of the analysed 396 microbiologically unconfirmed hospital cases, 40.4% were clinically diagnosed EM infections (Fig. 2). This finding indicates that the actual annual number of clinically diagnosed EM is probably a few hundred higher than what Avohilmo illustrates: some EM cases are missed because they are only reported to Hilmo and some are missed because they are diagnosed and treated in the occupational and private health care. As a limitation of this study, we do not have actual LB case numbers from the occupational or private health care.

Despite the serological result, around one third of the analysed 396 microbiologically unconfirmed hospital cases were clinically diagnosed LB (other than EM) (Fig. 2). According to the patient records, neurological symptoms and joint complaints were the most often mentioned manifestations leading to LB diagnosis and treatment, but in some cases, the clinical reasoning did not become fully clear to us. However, we cannot assess the validity of these diagnoses retrospectively, but it is noteworthy that a relatively big proportion of LB cases (other than EM) seem to be diagnosed clinically due to suggestive symptoms and, at the most, some reactivity in the serology. The heterogeneity of Hilmo stresses the need for clear, uniform LB case definitions, improved LB diagnostics, and increased awareness among physicians to improve the LB surveillance in Finland.

The lack of data from the occupational and private health care cause some underestimation of LB cases. In 2014, 13.3% of microbiologically confirmed LB cases could not be linked to any records in Avohilmo or Hilmo (Fig. 3). The most logical explanation would be that these cases derive either from occupational or private health care where Avohilmo and Hilmo are not used. A small proportion of these 226 cases could be such cases where laboratory diagnostics for LB is requested in the primary health care but despite the positive finding, other ICD-10 code than “A69.2” is used in Avohilmo. However, in the case of microbiologically confirmed LB cases, ~ 13% could roughly represent the utilization of occupational and private health care. This proportion likely varies between regions depending on the availability of the private health care.

This register-linkage allowed the assessment of case numbers and frequency of clinical diagnoses of LB in the study year 2014, and thereby also illustrated the reporting practices considering LB in Finland. Since this study, Avohilmo has been introduced in the routine surveillance of LB next to laboratory-based surveillance of microbiologically confirmed LB (NIDR) including LNB (available in Finnish in: Borrelioosin seuranta Suomessa. NIHW [25]). Avohilmo is updated on a weekly basis reflecting the epidemiological situation of LB infections and the seasonal variation of tick exposure timely. As a register for microbiologically confirmed disseminated LB infections, NIDR is suitable for the long-range temporal and geographical distribution analyses concerning LB incidence in Finland. Overall, the LB surveillance based on both registers jointly gives a comprehensive picture of LB incidence in our country considering both the early stage EM infections as well as the disseminated LB infections.

## Conclusions

The Register for Primary Health Care visits (Avohilmo) was found to be useful in monitoring clinically diagnosed EM infections, whereas the National Hospital Discharge Register (Hilmo) did not seem to improve surveillance of disseminated LB in Finland. Current laboratory-based surveillance (NIDR) of microbiologically confirmed LB cases provides better data on LB incidence of disseminated infection, including LNB. Since this study, Avohilmo was introduced in the routine surveillance of LB in Finland next to laboratory-based surveillance of disseminated LB.

We found a good compliance with the recommended treatment guidelines of clinically diagnosed LB in the primary health care. However, it remains to be analysed whether the prescribed dosing and the duration of the treatment for clinically diagnosed LB also follows the recommended guidelines.

## Data Availability

The data of this study are available from the NIHW, but restrictions apply to the availability of these data, which were used under license for the current study, and so are not publicly available. Data are however available from the authors upon request and with permission of the NIHW.

## Abbreviations

Avohilmo: The Register for Primary Health Care visits
EM: Erythema migrans
GP: General practitioner
Hilmo: the National Hospital Discharge Register
HD: Hospital district
ICD-10: the International Classification of Diseases, revision 10
LB: Lyme borreliosis
LNB: Lyme neuroborreliosis
NIDR: the National Infectious Diseases Register
NIHW: the National Institute for Health and Welfare

## References

[1] Stanek G, Wormser GP, Gray J, Strle F (2012). Lyme borreliosis. Lancet..

[2] Stanek G, Fingerle V, Hunfeld KP, Jaulhac B, Kaiser R, Krause A (2011). Lyme borreliosis: clinical case definitions for diagnosis and management in Europe. Clin Microbiol Infect.

[3] Shapiro ED (2014). Clinical practice. Lyme disease. N Engl J Med.

[4] Mead PS (2015). Epidemiology of Lyme disease. Infect Dis Clin N Am.

[5] Sykes RA, Makiello P. An estimate of Lyme borreliosis incidence in Western Europe. J Public Health. 2017;39(1):74–81. 10.1093/pubmed/fdw017 26966194

[6] Bennet L, Halling A, Berglund J (2006). Increased incidence of Lyme borreliosis in southern Sweden following mild winters and during warm, humid summers. Eur J Clin Microbiol Infect Dis.

[7] Smith R, Takkinen J. Lyme borreliosis: Europe-wide coordinated surveillance and action needed? Euro Surveill 2006;11(6):E060622.1. 10.2807/esw.11.25.02977-en 16819127

[8] Hubálek Z (2009). Epidemiology of Lyme borreliosis. Curr Probl Dermatol.

[9] Hofhuis A, Harms M, Bennema S, van den Wijngaard CC, van Pelt W (2015). Physician reported incidence of early and late Lyme borreliosis. Parasit Vectors.

[10] Theel ES (2015). Tickborne Borrelia Infections: Beyond Just Lyme Disease. Clin Lab Med.

[11] Lindgren E, Jaenson TG, Menne B (2006). Lyme borreliosis in Europe: influences of climate and climate change, epidemiology, ecology and adaptation measures.

[12] Dessau RB, Espenhain L, Mølbak K, Krause TG, Voldstedlund M. Improving national surveillance of Lyme neuroborreliosis in Denmark through electronic reporting of specific antibody index testing from 2010 to 2012. Euro Surveill. 2015;20(28):21184. 10.2807/1560-7917.es2015.20.28.21184 26212143

[13] Dahl V, Wisell KT, Giske CG, Tegnell A, Wallensten A. Lyme neuroborreliosis epidemiology in Sweden 2010 to 2014: clinical microbiology laboratories are a better data source than the hospital discharge diagnosis register. Euro Surveill. 2019;24(20):1800453. 10.2807/1560-7917.ES.2019.24.20.1800453 PMC6530252 31115310

[14] Wilking H, Stark K (2014). Trends in surveillance data of human Lyme borreliosis from six federal states in eastern Germany, 2009-2012. Ticks Tick Borne Dis.

[15] Chmielewska-Badora J, Moniuszko A, Żukiewicz-Sobczak W, Zwoliński J, Piątek J, Pancewicz S (2012). Serological survey in persons occupationally exposed to tick-borne pathogens in cases of co-infections with Borrelia burgdorferi, Anaplasma phagocytophilum, Bartonella spp. and Babesia microti. Ann Agric Environ Med.

[16] Vandenesch A, Turbelin C, Couturier E, Arena C, Jaulhac B, Ferquel E, et al. Incidence and hospitalisation rates of Lyme borreliosis, France, 2004 to 2012. Euro Surveill. 2014;19(34):20883. 10.2807/1560-7917.es2014.19.34.20883 25188613

[17] Sajanti E, Virtanen M, Helve O, Kuusi M, Lyytikäinen O, Hytönen J (2017). Lyme Borreliosis in Finland, 1995-2014. Emerg Infect Dis.

[18] Furu K, Wettermark B, Andersen M, Martikainen JE, Almarsdottir AB, Sørensen HT (2010). The Nordic countries as a cohort for pharmacoepidemiological research. Basic Clin Pharmacol Toxicol.

[19] Eliassen KE, Berild D, Reiso H, Grude N, Christophersen KS, Finckenhagen C (2017). Incidence and antibiotic treatment of erythema migrans in Norway 2005-2009. Ticks Tick Borne Dis..

[20] Van den Wijngaard CC, Hofhuis A, Simões M, Rood E, van Pelt W, Zeller H, et al. Surveillance perspective on Lyme borreliosis across the European Union and European Economic Area. Euro Surveill. 2017;22(27):30569. 10.2807/1560-7917.ES.2017.22.27.30569 PMC5508331 28703098

[21] Lindgren E, Andersson Y, Suk JE, Sudre B, Semenza JC (2012). Public health. Monitoring EU emerging infectious disease risk due to climate change. Science..

[22] Letrilliart L, Ragon B, Hanslik T, Flahault A (2005). Lyme disease in France: a primary care-based prospective study. Epidemiol Infect.

[23] Hytönen J, Hartiala P, Oksi J, Viljanen MK (2008). Borreliosis: recent research, diagnosis, and management. Scand J Rheumatol.

[24] Borchers AT, Keen CL, Huntley AC, Gershwin ME (2015). Lyme disease: a rigorous review of diagnostic criteria and treatment. J Autoimmun.

[25] Borrelioosin seuranta Suomessa. https://thl.fi/fi/web/infektiotaudit/taudit-ja-mikrobit/bakteeritaudit/borrelia/borrelioosin-seuranta?p_p_id=56_INSTANCE_dBI4l8nyV0vb&p_p_lifecycle=0&p_p_state=normal&p_p_mode=view&p_p_col_id=column-2-2-1&p_p_col_count=1. Accessed 9 Oct 2020.

